# Disrupted brain network functional dynamics and hyper‐correlation of structural and functional connectome topology in patients with breast cancer prior to treatment

**DOI:** 10.1002/brb3.643

**Published:** 2017-02-06

**Authors:** Shelli R. Kesler, Marjorie Adams, Melissa Packer, Vikram Rao, Ashley M. Henneghan, Douglas W. Blayney, Oxana Palesh

**Affiliations:** ^1^ Department of Neuro‐oncologyUniversity of Texas MD Anderson Cancer CenterHoustonTXUSA; ^2^ Department of Psychiatry and Behavioral SciencesStanford University School of MedicineStanfordCAUSA; ^3^ School of NursingUniversity of Texas at AustinAustinTXUSA; ^4^ Division of Medical OncologyStanford University School of MedicineStanfordCAUSA

**Keywords:** brain, cancer, cognition, connectome, fMRI, MRI, neuroimaging

## Abstract

**Introduction:**

Several previous studies have demonstrated that cancer chemotherapy is associated with brain injury and cognitive dysfunction. However, evidence suggests that cancer pathogenesis alone may play a role, even in non‐CNS cancers.

**Methods:**

Using a multimodal neuroimaging approach, we measured structural and functional connectome topology as well as functional network dynamics in newly diagnosed patients with breast cancer. Our study involved a novel, pretreatment assessment that occurred prior to the initiation of any cancer therapies, including surgery with anesthesia. We enrolled 74 patients with breast cancer age 29–65 and 50 frequency‐matched healthy female controls who underwent anatomic and resting‐state functional MRI as well as cognitive testing.

**Results:**

Compared to controls, patients with breast cancer demonstrated significantly lower functional network dynamics (*p* = .046) and cognitive functioning (*p* < .02, corrected). The breast cancer group also showed subtle alterations in structural local clustering and functional local clustering (*p* < .05, uncorrected) as well as significantly increased correlation between structural global clustering and functional global clustering compared to controls (*p* = .03). This hyper‐correlation between structural and functional topologies was significantly associated with cognitive dysfunction (*p* = .005).

**Conclusions:**

Our findings could not be accounted for by psychological distress and suggest that non‐CNS cancer may directly and/or indirectly affect the brain via mechanisms such as tumor‐induced neurogenesis, inflammation, and/or vascular changes, for example. Our results also have broader implications concerning the importance of the balance between structural and functional connectome properties as a potential biomarker of general neurologic deficit.

## 1 Introduction

Cancer and/or its therapies appear to be associated with brain injury that results in cognitive impairment. Several cross‐sectional and longitudinal studies have demonstrated abnormalities in brain structure and function, particularly in chemotherapy‐treated patients and survivors (D'Agata et al., 2013; Deprez et al., 2014; Jung et al., 2016; Kesler & Blayney, 2015; Lepage et al., 2014; Nudelman et al., 2014; Simo et al., 2015; Stouten‐Kemperman et al., 2014). However, many patients demonstrate differences in brain structure and function prior to chemotherapy suggesting that cancer pathogenesis, surgery/anesthesia, disease burden, host‐related, and/or other factors may contribute to early brain changes (Cimprich et al., 2010; McDonald, Conroy, Ahles, West, & Saykin, 2012; Menning et al., 2015; Sato et al., 2015; Scherling, Collins, Mackenzie, Bielajew, & Smith, 2012; Scherling, Collins, Mackenzie, Lepage, et al., 2012).

Interestingly, while studies suggest that chronic effects of cancer and its treatments are characterized by lower brain functional activation (Kesler, Kent, & O'Hara, 2011; de Ruiter et al., 2011), evaluation of newly diagnosed patients consistently indicates hyper‐activation (McDonald et al., 2012; Scherling, Collins, Mackenzie, Bielajew, & Smith, 2011). The reasons for this pattern are unclear but early hyper‐activation may represent disease‐related brain injury that disrupts appropriate neural resource allocation and/or functional dynamics. Support for our hypothesis includes a postsurgery/prechemotherapy study of patients with breast cancer that demonstrated disrupted scale‐free functional dynamics (Churchill et al., 2015). Additionally, we have previously noted a potential alteration in the relationship between structural and functional connectome properties in long‐term survivors of breast cancer (Kesler, Watson, & Blayney, 2015) that may result in restricted flexibility of the functional network (Wirsich et al., 2016). However, to date, no studies have evaluated the relationship between structural and functional connectomes in the same cohort of patients with breast cancer.

The connectome is a mathematical representation of the brain network comprised of regions (nodes) and connections (edges) between regions. This approach to evaluate brain connectivity relies on graph theory, which is the study of objects and their connections. Connectomes display a “small‐world” organization wherein specialized groups or clusters of neurons are highly connected to each other while being economically connected to other clusters (Bassett and Bullmore, 2006). Thus, connectome properties provide unique insights regarding both the integration and segregation of the brain network.

To date, pretreatment neuroimaging studies have involved a postsurgery/prechemotherapy baseline. Given evidence that surgery and/or anesthesia may be associated with cognitive and brain changes (Chen, Miaskowski, Liu, & Chen, 2012; Sato et al., 2015), the effects of cancer alone remain unclear. A recent study by Patel et al. (2015) demonstrated significantly reduced cognitive function in patients with breast cancer compared to healthy controls prior to initiation of any treatment, including surgery. These cognitive impairments were associated with elevated pro‐inflammatory cytokine levels. The effects of peripheral inflammation on cognition have been shown to be mediated by changes in the brain (Harrison, Doeller, Voon, Burgess, & Critchley, 2014). Therefore, it is likely that disruptions of brain structure and/or function also exist at this early, pretreatment stage of breast cancer. These disruptions may parallel, at least in part, those noted following cancer treatments given that inflammation is a candidate mechanism underlying cancer pathogenesis as well as chemotherapy‐related effects on the brain (Janelsins, Kesler, Ahles, & Morrow, 2014; Patel et al., 2015).

As part of our prospective, longitudinal study of cognition in breast cancer, we evaluated newly diagnosed patients prior to any treatment, including surgery with anesthesia. In this initial study, we aimed to compare brain structure and function, including functional dynamics, at our pretreatment baseline. We hypothesized that patients would demonstrate lower functional connectivity and dynamics compared to controls, based on the previous studies noted above that obtained postsurgery/prechemotherapy baselines. We also hypothesized that the relationship between structural and functional connectome topologies would be altered in the breast cancer group compared to controls, based on our previous work in breast cancer survivors, as described above. We employed a multimodal neuroimaging approach including advanced methods that emphasize multivariate, brain network connectivity.

## 2 Methods

### 2.1 Participants

At the time of this study, we had enrolled 74 women aged 29–65 years with newly diagnosed primary breast cancer who had completed their initial study visit prior to any treatment (surgery, chemotherapy, and breast radiation therapy). We also enrolled 50 healthy female controls frequency matched for age, education, and menopausal status (Table 1). Patients with breast cancer were recruited at the Stanford Cancer Institute. All consecutive patients who met study eligibility criteria were approached. Healthy controls were recruited via local media advertisements in northern California communities. There were 156 participants screened; 32 were excluded or declined to participate. Participants were excluded for psychiatric, neurologic, or comorbid medical conditions that are known to affect cognitive function as well as any major sensory deficits (e.g., blindness). Participants were also required to be fluent in English sufficient for valid cognitive testing. The Stanford University Institutional Review Board approved this study, and all participants provided informed consent.

**Table 1:** Demographic and medical variables

	Breast cancer, *N* = 74	Healthy controls *N* = 50	*F*/Chi Sq.	*p*
Age	49.8 (9.3)	49.7 (10.0)	0.057	.95
Age range	29–66	26–64		
Education (years)	17.0	17.5	−1.083	.28
Minority status	33%	20%	2.46	.117
Postmenopausal	45%	40%	0.328	.567
Disease stage at diagnosis (0, I, II, III)	6%, 35%, 47%, 11%			
Days since diagnosis	38 (26)			
Estrogen receptor positive	89%			
Progesterone receptor positive	75%			
Estrogen/progesterone receptor positive	75%			
HER2 positive	24%			
BRCA (BRCA1 positive, BRCA2 positive)	9%, 9%			

HER2, human epidermal growth factor receptor 2; BRCA, breast cancer susceptibility.

### 2.2 Cognitive performance

Trained research staff administered the following neuropsychological tests to all participants: Comprehensive Trail Making Test (CTMT) (Moses, 2004), Controlled Oral Word Association (COWA) (Ruff, Light, Parker, & Levin, 1996), and the Rey Auditory Verbal Learning Test (RAVLT) (Schmidt, 2012). Participants were also administered domain‐specific self‐report measures, including the Clinical Assessment of Depression (CAD) (Aghakhani & Chan, 2007), a measure of depression, anxiety, and fatigue, the Behavioral Rating Inventory of Executive Function for Adults (BRIEF) (Roth, Isquith, & Gioia, 2005), and the Prospective and Retrospective Memory Questionnaire (PRMQ) (Crawford, Henry, Ward, & Blake, 2006; Crawford, Smith, Maylor, Della Sala, & Logie, 2003). Testing required approximately 1 hr. Raw scores were used for RAVLT, while age‐adjusted raw scores were used for COWA and T scores for CTMT, based on these tests' normative data.

### 2.3 Neuroimaging acquisition

Participants were included in this study even if they had MRI contraindications; 65 of the 74 patients with breast cancer and all 50 of the controls underwent MRI. Neuroimaging data were acquired using a GE Discovery MR750 3.0 Tesla whole body scanner (GE Medical Systems) on the same day as the cognitive testing session. Resting‐state functional magnetic resonance imaging (rsfMRI) data were acquired while participants rested in the scanner with their eyes closed. We used a T2*‐weighted gradient echo spiral pulse sequence (Glover & Law, 2001) with the following parameters: relaxation time = 2,000 ms, echo time = 30 ms, flip angle = 89° and 1 interleave, field of view = 200, matrix = 64 × 64, in‐plane resolution = 3.125. Number of volumes collected was 216, scan time = 7:12. An automated high‐order shimming method was used to reduce field inhomogeneity.

We also acquired a high‐resolution, 3D inversion‐recovery prepared fast spoiled gradient echo T1‐weighted anatomical MRI scan with the following parameters: TR = minimum, TE = minimum, flip = 11 degrees, inversion time = 300 ms, bandwidth = ±31.25 kHz, field of view = 24 cm, phase field of view = 0.75, slice thickness = 1.5 mm, 125 slices, 256 × 256 at 1 excitation, scan time = 4:26. Some participants also underwent diffusion tensor imaging if time allowed (total scan time = 30 min or less). These data are not reported here. Neuroimaging data were visually inspected for quality prior to any preprocessing.

### 2.4 Functional connectome construction

Functional connectivity preprocessing was performed using Statistical Parametric Mapping 8 (SPM8, RRID:SCR_007037) and CONN Toolbox (RRID:SCR_009550) as previously described (Kesler & Blayney, 2015; Kesler et al., 2013, 2014). Successful normalization was confirmed via visual inspection using the check registration function in SPM8 and in‐house software that creates whole volume slice montages. Artifact correction included wavelet despiking (Patel et al., 2014). Correlation coefficients were calculated between rsfMRI time courses for each pair of 90 Automated Anatomical Labeling Atlas (AAL) (Tzourio‐Mazoyer et al., 2002) regions of interest (ROIs) and then normalized using Fisher's r‐z transformation. Realignment motion parameters were included as covariates and images with excessive motion/signal artifact were excluded. The resulting z‐score connectivity matrices were thresholded to minimum connection density and then submitted to graph theoretical analysis using our Brain Networks Toolbox (https://github.com/srkesler/bnets.git, RRID:SCR_014788) as well as Brain Connectivity Toolbox (Rubinov & Sporns, 2010) (RRID:SCR_004841) implemented in MATLAB v2014b. We focused on the clustering coefficient considering our previous findings (Bruno, Hosseini, & Kesler, 2012; Kesler, Gugel, Huston‐Warren, & Watson, 2016; Kesler et al., 2015). Clustering coefficient reflects the ratio of actual to possible connections between a node's neighbors and is therefore a measure of network segregation (Rubinov & Sporns, 2010). We examined both global clustering (mean clustering coefficient across all nodes) as well as local clustering (nodal clustering).

### 2.5 Functional network dynamics

We evaluated the temporal dynamics of the functional network by calculating the rescaled range Hurst exponent (Hurst, 1951) for all 90 ROIs, corrected for small sample bias. The windowing function was based on a data‐derived natural number that possessed the largest number of divisors among all natural numbers in the time series interval. The Hurst exponent quantifies how correlated a time series is with itself, or how well it reflects elements of the baseline signal from both the recent and remote past. This autocorrelative property is referred to as “long memory” (Churchill et al., 2015; Ciuciu, Varoquaux, Abry, Sadaghiani, & Kleinschmidt, 2012; He, 2011).

### 2.6 Structural connectome construction

Gray matter maps were obtained using voxel‐based morphometry (VBM). Images were first manually reoriented to the anterior and posterior commissures then realigned, segmented into tissue compartments, spatially normalized to a template using diffeomorphic anatomical registration through exponentiated lie algebra (DARTEL), and modulated and smoothed (12 mm full width, half maximum kernel) using the VBM8 Toolbox within SPM8 (Kurth, Gaser, & Luders, 2015). Successful normalization was confirmed via visual inspection using the check registration function in SPM8 and whole volume slice montages as well as with the check sample homogeneity function in VBM8 Toolbox.

Gray matter covariance networks were constructed for each patient using an innovative similarity‐based extraction method (Tijms, Series, Willshaw, & Lawrie, 2012). Network nodes were defined by 3 × 3 × 3 voxel cubes spanning the entire gray matter volume (mean network size = 8,525 ± 49 nodes). Each node therefore contained 27 gray matter volume values and a correlation matrix was calculated across all pairs of nodes taking into account the sum over the product of the differences between the cubes' values at each voxel location as well as the cubes' average values (Tijms et al., 2012). The correlation matrices were thresholded to minimum connection density and evaluated using graph theoretical analysis as described above. For local analysis, nodes were assigned one of the 90 AAL labels based on the node's voxel coordinates. Nodal clustering was calculated for each node as the average clustering coefficient across all nodes with the same AAL label as previously described (Tijms et al., 2012).

### 2.7 Statistical analyses

Between‐group differences in cognitive test scores and clustering coefficients were calculated using the general linear model, covarying for minority status and CAD score. Structural clustering was additionally covaried for structural connectome size (i.e., number of nodes) given that this varied between individuals. Local clustering and cognitive test score models were corrected for false discovery rate (FDR). Hurst long memory was not normally distributed and was therefore evaluated using Wilcoxon rank test for both global and local effects (with FDR correction for local effects).

Within both groups separately, the relationships among functional clustering and structural clustering and Hurst long memory were explored using two‐tailed correlations. Differences in correlations between the groups were evaluated using two‐tailed Fisher r‐to‐z transformation. To examine the effect of the relationship between structural clustering and functional clustering on cognitive performance, we conducted a principle component analysis (PCA) on structural and functional clustering coefficients across all participants.

Within the breast cancer group, exploratory, two‐tailed correlations were performed to examine the relationships among education level, age, disease stage, days since diagnosis, brain metrics, and cognition. Exploratory, two‐tailed t‐ or rank tests were calculated to determine whether menopausal status or tumor pathology contributed to neurobiologic status and/or cognitive performance. Tumor pathology included hormone receptor (estrogen/progesterone), human epidermal growth factor receptor 2, and breast cancer susceptibility status obtained from the patient's medical record. Only the brain metrics and cognitive tests that were significantly different between groups were examined. To reduce the number of comparisons, a composite of the significant cognitive test scores was computed using the Mahalanobis distance, which was then log transformed (Mahalanobis, 1936; Menning et al., 2015; Stouten‐Kemperman et al., 2015).

All statistical analyses were conducted in the R statistical package (R Foundation, RRID:SCR_001905).

## 3 Results

### 3.1 Cognitive performance

The breast cancer group demonstrated lower scores on all cognitive tests except for CTMT trial 3. Of the 10 cognitive measures, the RAVLT total recall and interference trials, CTMT trial 1 and COWA scores were significant and survived FDR correction (Table 2). CAD was not a significant covariate in these models (*p* > .19), but minority status was for RAVLT interference and COWA (*p* < .04).

**Table 2:** Cognitive and self‐report measures

	Breast cancer (*N* = 74)	Healthy controls (*N* = 50)	*F*/Chi Sq.	*p*	*p* (FDR corrected)
RAVLT total recall	52.5 (8.6)	56.1 (7.6)	7.64	.01	.02
RAVLT interference	5.82 (1.8)	6.76 (1.8)	8.21	.01	.02
RAVLT delayed recall	10.9 (2.7)	11.6 (2.2)	3.07	.08	.12
CTMT 1	50.7 (7.3)	55.5 (9.7)	9.16	.003	.02
CTMT 2	52.7 (10.6)	54.2 (10.4)	0.45	.50	.56
CTMT 3	50.1 (8.2)	50.1 (10.1)	0.01	.91	.91
CTMT 4	54.8 (10.1)	56.5 (10.1)	0.46	.50	.56
CTMT 5	50.6 (8.8)	54.0 (9.5)	4.35	.04	.07
COWA	42.5 (13.0)	49.5 (12.8)	7.44	.01	.02
BRIEF GEC^a^	51.3 (9.2)	45.3 (9.8)	0.74	.39	
PRMQ^a^	36.7 (8.8)	32.8 (8.2)	0.89	.35	
CAD^a^	52.0 (9.8)	43.7 (9.6)	22.8	<.0001	

RAVLT, Rey Auditory Verbal Learning Test; CTMT, Comprehensive Trail Making Test; COWA, Controlled Oral Word Association; BRIEF GEC, Behavioral Rating Inventory of Executive Function Global Executive Composite; PRMQ, Prospective and Retrospective Memory Questionnaire; CAD, clinical assessment of depression; FDR, false discovery rate.

a Higher scores on the BRIEF, PRMQ, CAD = elevated symptoms. Higher scores on all other measures = better performance.

### 3.2 Self‐ratings

The breast cancer group showed elevated psychological distress as measured by the CAD compared to controls (*p* < .0001, Table 2). Minority status was nonsignificant in the model (*p* = .99). There were no significant differences in subjective executive or memory function (Table 2). CAD was a very significant covariate in these models (*p* < .0001), but minority status was not (*p* > .87).

### 3.3 Functional network clustering coefficient

**Figure 1:**
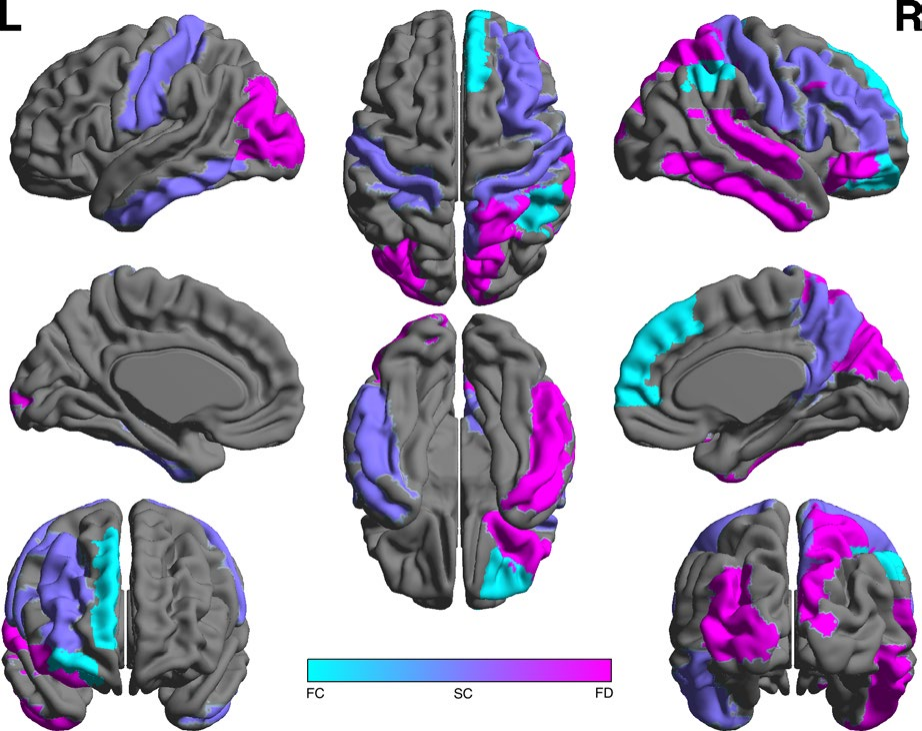
Local differences in brain network metrics. Compared to controls, patients with breast cancer showed altered functional clustering (FC, cyan) in right inferior parietal lobe, right middle inferior orbital frontal gyrus, and right medial superior frontal gyrus (*p* < .05, uncorrected). The breast cancer group showed altered structural clustering (SC, blue) in right inferior and middle frontal gyri, bilateral postcentral gyri, right precuneus, and left inferior temporal gyrus (*p* < .05, uncorrected). Functional dynamics as measured by Hurst exponent (FD, magenta) was lower in patients with breast cancer compared to controls in right inferior orbital gyrus, left middle occipital gyrus, right parietal lobule, right cuneus, right superior temporal gyrus, and right inferior temporal gyrus (*p* < .05, uncorrected)

No significant difference was observed between the groups in global clustering (*p* = .19, Table 3). However, the breast cancer group showed significantly altered local clustering in several frontal and parietal regions, but these did not survive FDR correction (Figure 1). Minority status and CAD were nonsignificant covariates (*p* > .51).

**Table 3:** Brain network metrics

	Breast cancer (*N* = 65)	Healthy controls (*N* = 50)	*F*	*p*
Functional connectome global clustering coefficient	0.54 (0.03)	0.53 (0.03)	1.78	.19
Structural connectome global clustering coefficient	0.69 (0.005)	0.70 (0.005)	0.26	.61
Structural connectome size	8,521 (48)	8,531 (49)	0.01	.92
Functional network dynamics (Hurst exponent)	0.19 (0.10)	0.22 (0.11)	1,271	.046

### 3.4 Structural network clustering coefficient

No significant group difference was found in global clustering (*p* = .61, Table 3). Again, local clustering was significantly altered in frontal and parietal as well as temporal regions in the breast cancer group but not after FDR correction (Figure 1). Minority status and CAD were not significant covariates (*p* > .53).

### 3.5 Functional network dynamics

The breast cancer group demonstrated significantly lower Hurst long memory compared to controls (*p* = .046, Table 3). This primarily involved distributed, right lateralized regions (Figure 1). Hurst long memory was not correlated with CAD or minority status in either group (*p* > .33).

### 3.6 Brain structure and function relationships

**Figure 2:**
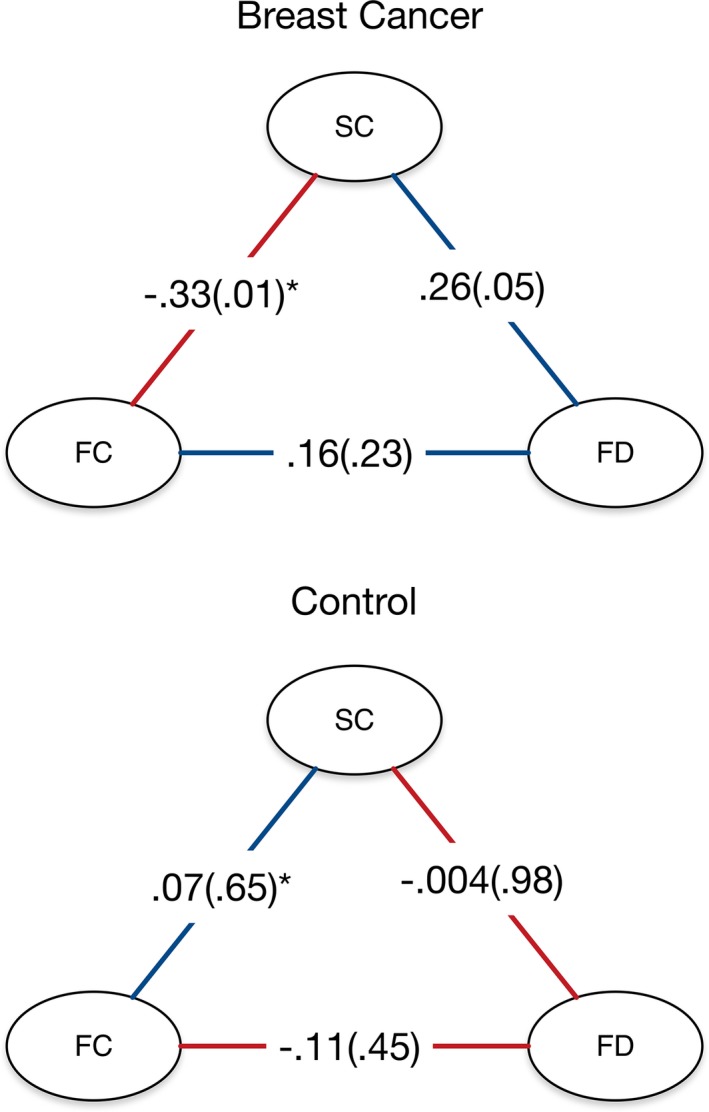
Correlations between brain network metrics. The breast cancer group demonstrated a significant negative correlation between structural and functional clustering as well as a significant positive correlation between functional clustering and Hurst exponent. Values are shown as r(*p*). SC, structural connectome clustering; FC, functional connectome clustering; FD, functional dynamics (Hurst exponent). *The group difference between these correlations was significant (*p* = .03)

Structural clustering and functional clustering as well as functional clustering and Hurst long memory were significantly associated, although only in the breast cancer group (Figure 2). Specifically, structural clustering and functional clustering were inversely correlated (r = −0.33, *p* = .01) and structural clustering was directly correlated with Hurst long memory (r = 0.26, *p* = .050). The difference in correlation between the groups was significant for structural clustering and functional clustering (z = 2.21, *p* = .03) but not for structural clustering and Hurst long memory (z = 1.4, *p* = .16). We examined only the first PCA component, which accounted for 59% of the variance. This component weighted functional clustering negatively and structural clustering positively. Component scores were not correlated with CAD or minority status (*p* > .31).

### 3.7 Brain and cognition

**Figure 3:**
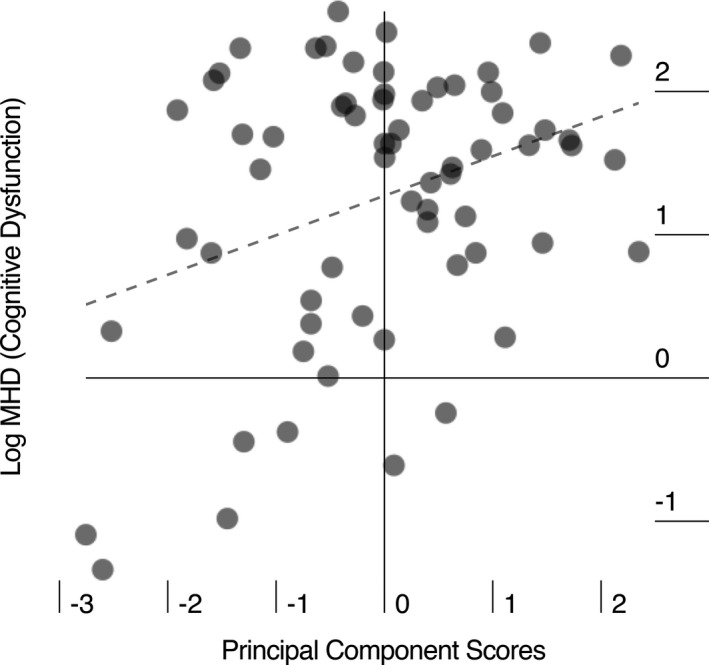
Relationship of structural and functional principal component and cognitive function in patients with breast cancer. Greater cognitive dysfunction was associated with greater overlap between structural and functional connectome clustering (r = 0.34, *p* = .005). MHD, Mahalanobis distance; higher MHD = greater cognitive dysfunction

In the breast cancer group, Mahalanobis distance was significantly correlated with PCA component scores (Figure 3, r = 0.34, *p* = .005) and moderately associated with Hurst long memory (r = −0.21, *p* = .09). Higher Mahalanobis distance indicates higher deviance of cognitive scores from the control group and therefore greater cognitive dysfunction.

### 3.8 Disease, demographics, brain, and cognition

There were no differences in Hurst long memory, PCA component or Mahalanobis distance related to disease stage, tumor pathology, or menopausal status. There were also no significant correlational effects of demographic variables or days since diagnosis on brain or cognition.

## 4 Discussion

Our study replicates findings reported by Patel et al. (2015) in that we observed cognitive impairment in newly diagnosed patients with breast cancer at a pretreatment evaluation. Unlike most previous studies, patients enrolled in this study had yet to undergo any cancer treatment, including any surgery with general anesthesia. Importantly, our study provides novel information by demonstrating neural biomarkers of cognitive dysfunction associated with breast cancer.

Using a multimodal, multivariate approach, we examined the topology of both structural and functional connectomes as well as the temporal dynamics of the functional network. We demonstrated that functional dynamics were significantly lower in patients with breast cancer compared to controls. This method is based on the theory that the brain resides in a state of “criticality” allowing it to adapt quickly to new situations. A critical state system is characterized by spatial and temporal correlations that show long memory, which theoretically represents the brain network's ability to keep relevant information readily available, allowing it to respond dynamically (Ciuciu et al., 2012; He, 2011).

Our findings indicate that the adaptability of functional networks is disrupted even prior to initiation of cancer treatments, including surgery. Conversely, Churchill et al. (2015) observed higher Hurst exponent in patients with breast cancer compared to healthy controls (postsurgery/prechemotherapy). They also noted relationships between Hurst long memory and psychological distress, which we did not. However, their study involved a task‐fMRI paradigm, whereas ours involved resting‐state fMRI. Hurst exponent is highest during resting state and tends to decrease with increased task load (He, 2011; He, Zempel, Snyder, & Raichle, 2010). Therefore, the Churchill et al. findings may suggest a deficit in task‐modulated suppression of long memory. This would be consistent with other studies demonstrating reduced task‐related functional deactivation in patients with breast cancer (Cimprich et al., 2010; Kesler, Bennett, Mahaffey, & Spiegel, 2009).

We have previously observed altered structural and functional clustered connectivity in our cross‐sectional studies of chemotherapy‐treated, long‐term breast cancer survivors (Bruno et al., 2012; Hosseini, Koovakkattu, & Kesler, 2012; Kesler et al., 2015). In the present study, we demonstrated only very subtle disruption of local clustered connectivity in pretreatment patients. Taken together, these findings suggest that this injury may begin quite early in the disease course but is more affected chronically and/or by adjuvant treatments. Regional clustering and Hurst exponent results indicate a widespread effect, consistent with previous studies (Deprez et al., 2012; Kesler et al., 2015). Frontal, parietal, and temporal areas were altered in the breast cancer group compared to controls, consistent with deficits in memory, executive function, and verbal fluency. It will be important to evaluate how these regional effects change in patients after adjuvant treatments. It should also be noted that regional differences did not survive correction for multiple comparisons and may therefore be spurious.

These potential alterations in clustered connectivity involved both functional and structural networks. Unlike most previous studies of gray matter structural covariance networks, the similarity‐based extraction method we applied resulted in individual level networks that allowed us to examine correlations with functional connectomes. Gray matter structural covariance networks are believed to reflect underlying axonal connections as well as common genetic, neurotrophic, and neuroplastic processes (Alexander‐Bloch, Giedd, & Bullmore, 2013; Mechelli, Friston, Frackowiak, & Price, 2005). Our group and others have previously demonstrated, in healthy adults, that structural covariance networks are consistent with intrinsic functional networks with respect to connectivity pattern, although not in all brain regions (Damoiseaux & Greicius, 2009; Hosseini & Kesler, 2013).

Additionally, the *topologies* of structural and functional networks, as measured by connectome properties such as clustering coefficient, tend to be uncorrelated (Caeyenberghs, Leemans, Leunissen, Michiels, & Swinnen, 2013; Hosseini & Kesler, 2013). Consistently, in the present study, we found no significant relationship between structural and functional clustering coefficients in healthy controls. Additionally, there was little overlap between functional and structural regional clustering differences between the groups (Figure 1). The meaning of this lack of correlation in healthy individuals is currently unclear but may indicate the presence of indirect functional connections, functional gating, and/or interregional distance (Deco & Corbetta, 2011; Honey et al., 2009; Stam et al., 2016).

Unlike controls, the breast cancer group showed a significant inverse relationship between structural clustering and functional clustering. In our previous studies of breast cancer survivors, we have noted a similar pattern in terms of group differences wherein structural connectome clustering was elevated in one study while functional connectome clustering was reduced compared to controls in a separate study (Bruno et al., 2012; Kesler et al., 2015). This may suggest a disease‐related disruption in the balance between structural and functional connectome organizations. The brain involves many opposing demands that might be represented by anticorrelated processes. However, the brain is organized to reconcile these demands (Rubinov & Sporns, 2010; Sporns & Honey, 2006), and therefore, hyper‐inverse correlation seems to suggest a disruption in the brain network's ability to balance competing systems. Accordingly, our principal component analysis inversely weighted structural clustering and functional clustering and these component scores were significantly correlated with cognitive dysfunction.

A review of connectome studies in Alzheimer's disease also suggests inverse relationships between structural and functional connectome topologies across single‐modality studies (Dai et al., 2015). Additionally, an effect of inverse structural and functional connectome relationship on behavioral phenotype was observed in a previous multimodal study of individuals with pervasive developmental disorder (Rudie et al., 2012). Additionally, hyper‐correlation of structural and functional connectome properties has been associated with temporal lobe epilepsy (Wirsich et al., 2016). Therefore, enhanced structure–function correlation seems likely to be a general effect of neurologic disorder rather than a cancer‐specific one.

One possible interpretation of our results is that increased structural clustering results in decreased flexibility of functional networks. This interpretation is supported by our finding that lower structural clustering was associated with lower functional adaptability (Hurst long memory). Additionally, lower Hurst long memory was moderately, though nonsignificantly correlated with greater cognitive dysfunction in patients with breast cancer. Brain structure is believed to constrain brain function (Deco & Corbetta, 2011) and an optimal balance between brain network stability and flexibility is required for learning (Hermundstad, Brown, Bassett, & Carlson, 2011a, 2011b).

Given the nature of correlation, the alternative interpretation that increased functional clustering decreases structural clustering is also possible. The functional hyper‐activation noted in postsurgery/prechemotherapy studies of patients with breast cancer may reflect abnormal, excitotoxic neural activity as observed in other neurologic syndromes (Palop & Mucke, 2010). We have previously demonstrated that chemotherapy upregulates neural activity and increases synaptic death in cultured neurons (Manchon et al., 2016). Perhaps these processes are also associated with aspects of cancer pathogenesis. Specifically, breast cancer tumors initiate neurogenesis and release nerve growth factor (Cole, Nagaraja, Lutgendorf, Green, & Sood, 2015; Pundavela et al., 2015; Zhao et al., 2014), which may result in aberrant CNS activity via peripheral innervation. Tumor aggressiveness has been associated with tumor‐related neurogenesis (Zhao et al., 2014). However, we were unable to detect any effects of disease severity or tumor pathology on brain connectivity or cognitive function. This may have been due to the imbalance in our sample with respect to disease stage and tumor markers, and therefore, further investigation is required.

As noted above, Patel et al. (2015) observed correlations between elevated cytokine levels and cognitive dysfunction in patients with breast cancer presurgery. Malignant tumors and their inflammatory response are also characterized by angiogenesis (Farnsworth, Lackmann, Achen, & Stacker, 2014; Folkman, 1971). A preliminary study by Ng et al. (2013) indicated elevated plasma vascular endothelial growth factor (VEGF), a common angiogenic factor, in patients with breast cancer following chemotherapy treatment, that was negatively correlated with cognition. VEGF is expressed in the brain and is believed to play an important role in neurodegeneration. However, the study did not evaluate baseline, pretreatment VEGF levels. Long‐term breast cancer survivors also have increased incidence of cerebral small vessel disease following cancer therapies (Koppelmans et al., 2015), but it is currently unknown if these vascular effects exist prior to treatment initiation. The potential role of these factors in cancer‐related cognitive impairment requires further investigation.

Stress is also associated with disrupted functional dynamics in patients with breast cancer, inflammatory response, and tumor progression (Churchill et al., 2015; Cole et al., 2015). Psychological distress, as measured by CAD, was significantly higher in the breast cancer group compared to controls, but was not a significant contributor to group differences in cognitive performance and was not correlated with Hurst long memory or structure–function component. Mean CAD score in the breast cancer group was in the clinically defined “normal range” for this measure (Aghakhani & Chan, 2007). Therefore, psychological distress may not have been sufficiently elevated to have a detectable impact on brain function. These findings provide further evidence that cognitive impairment in patients with breast cancer cannot be entirely explained by distress. We also noted that distress contributes primarily to self‐reported rather than objective cognitive function, consistent with previous studies (Wefel, Kesler, Noll, & Schagen, 2015).

In summary, prior to initiation of any treatments, including surgery with anesthesia, patients with newly diagnosed breast cancer showed disruption of intrinsic functional network dynamics and altered relationship between structural and functional connectome clustering. Our results provide important new insights regarding the effects of non‐CNS cancer on brain network organization with broader implications concerning the significance of the relationship between structural and functional connectome properties as a potential biomarker of neurologic deficit. This study, in combination with the previous literature in this area, suggests that these effects may represent a cumulative injury to the brain, beginning with cancer pathogenesis and then increasing in severity across subsequent cancer treatments. Further research is needed to address the limitations of this study including replication of structural connectomes using fiber tractography and targeted recruitment of patients to balance samples in terms of tumor pathology and disease severity.

## Conflict of interest

The authors have no conflicts of interest related to this research.

## References

[brb3643-bib-0001] Aghakhani, A. , & Chan, E. K. (2007). Test Reviews: Bracken, B. A., & Howell, K. (2004). Clinical Assessment of Depression. Odessa, FL: Psychological Assessment Resources. Journal of Psychoeducational Assessment, 25, 416–422.

[brb3643-bib-0002] Alexander‐Bloch, A. , Giedd, J. N. , & Bullmore, E. (2013). Imaging structural co‐variance between human brain regions. Nature Reviews Neuroscience, 14, 322–336. 23531697 10.1038/nrn3465 PMC4043276

[brb3643-bib-0101] Bassett, D. S. , & Bullmore, E. (2006). Small‐world brain networks. The neuroscientist, 12, 512–523. 17079517 10.1177/1073858406293182

[brb3643-bib-0003] Bruno, J. , Hosseini, S. M. , & Kesler, S. (2012). Altered resting state functional brain network topology in chemotherapy‐treated breast cancer survivors. Neurobiology of Diseases, 48, 329–338. 10.1016/j.nbd.2012.07.009 PMC3461109 22820143

[brb3643-bib-0004] Caeyenberghs, K. , Leemans, A. , Leunissen, I. , Michiels, K. , & Swinnen, S. P. (2013). Topological correlations of structural and functional networks in patients with traumatic brain injury. Frontiers in Human Neuroscience, 7, 726. 24204337 10.3389/fnhum.2013.00726 PMC3817367

[brb3643-bib-0005] Chen, M. L. , Miaskowski, C. , Liu, L. N. , & Chen, S. C. (2012). Changes in perceived attentional function in women following breast cancer surgery. Breast Cancer Research and Treatment, 131, 599–606. 21901384 10.1007/s10549-011-1760-3

[brb3643-bib-0006] Churchill, N. W. , Cimprich, B. , Askren, M. K. , Reuter‐Lorenz, P. A. , Jung, M. S. , Peltier, S. , & Berman, M. G. (2015). Scale‐free brain dynamics under physical and psychological distress: Pre‐treatment effects in women diagnosed with breast cancer. Human Brain Mapping, 36, 1077–1092. 25388082 10.1002/hbm.22687 PMC6869445

[brb3643-bib-0007] Cimprich, B. , Reuter‐Lorenz, P. , Nelson, J. , Clark, P. M. , Therrien, B. , Normolle, D. , … Welsh, R. C. (2010). Prechemotherapy alterations in brain function in women with breast cancer. Journal of Clinical and Experimental Neuropsychology, 32, 324–331. 19642048 10.1080/13803390903032537

[brb3643-bib-0008] Ciuciu, P. , Varoquaux, G. , Abry, P. , Sadaghiani, S. , & Kleinschmidt, A. (2012). Scale‐Free and Multifractal Time Dynamics of fMRI Signals during Rest and Task. Frontiers in Physiology, 3, 186. 22715328 10.3389/fphys.2012.00186 PMC3375626

[brb3643-bib-0009] Cole, S. W. , Nagaraja, A. S. , Lutgendorf, S. K. , Green, P. A. , & Sood, A. K. (2015). Sympathetic nervous system regulation of the tumour microenvironment. Nature Reviews Cancer, 15, 563–572. 26299593 10.1038/nrc3978 PMC4828959

[brb3643-bib-0010] Crawford, J. R. , Henry, J. D. , Ward, A. L. , & Blake, J. (2006). The Prospective and Retrospective Memory Questionnaire (PRMQ): Latent structure, normative data and discrepancy analysis for proxy‐ratings. British Journal of Clinical Psychology, 45, 83–104. 16480568 10.1348/014466505X28748

[brb3643-bib-0011] Crawford, J. R. , Smith, G. , Maylor, E. A. , Della Sala, S. , & Logie, R. H. (2003). The Prospective and Retrospective Memory Questionnaire (PRMQ): Normative data and latent structure in a large non‐clinical sample. Memory, 11, 261–275. 12908675 10.1080/09658210244000027

[brb3643-bib-0012] D'Agata, F. , Costa, T. , Caroppo, P. , Baudino, B. , Cauda, F. , Manfredi, M. , … Bisi, G. (2013). Multivariate analysis of brain metabolism reveals chemotherapy effects on prefrontal cerebellar system when related to dorsal attention network. EJNMMI Res, 3, 22. 23557152 10.1186/2191-219X-3-22 PMC3637083

[brb3643-bib-0013] Dai, Z. , Yan, C. , Li, K. , Wang, Z. , Wang, J. , Cao, M. , … He, Y. (2015). Identifying and Mapping Connectivity Patterns of Brain Network Hubs in Alzheimer's Disease. Cerebral Cortex, 25, 3723–3742. 25331602 10.1093/cercor/bhu246

[brb3643-bib-0014] Damoiseaux, J. S. , & Greicius, M. D. (2009). Greater than the sum of its parts: A review of studies combining structural connectivity and resting‐state functional connectivity. Brain Structure & Function, 213, 525–533. 19565262 10.1007/s00429-009-0208-6

[brb3643-bib-0015] Deco, G. , & Corbetta, M. (2011). The dynamical balance of the brain at rest. Neuroscientist, 17, 107–123. 21196530 10.1177/1073858409354384 PMC4139497

[brb3643-bib-0016] Deprez, S. , Amant, F. , Smeets, A. , Peeters, R. , Leemans, A. , Van Hecke, W. , … Sunaert, S. (2012). Longitudinal Assessment of Chemotherapy‐Induced Structural Changes in Cerebral White Matter and Its Correlation With Impaired Cognitive Functioning. Journal of Clinical Oncology, 30, 274–281. 22184379 10.1200/JCO.2011.36.8571

[brb3643-bib-0017] Deprez, S. , Vandenbulcke, M. , Peeters, R. , Emsell, L. , Smeets, A. , Christiaens, M. R. , … Sunaert, S. (2014). Longitudinal assessment of chemotherapy‐induced alterations in brain activation during multitasking and its relation with cognitive complaints. Journal of Clinical Oncology, 32, 2031–2038. 24868029 10.1200/JCO.2013.53.6219

[brb3643-bib-0018] Farnsworth, R. H. , Lackmann, M. , Achen, M. G. , & Stacker, S. A. (2014). Vascular remodeling in cancer. Oncogene, 33, 3496–3505. 23912450 10.1038/onc.2013.304

[brb3643-bib-0019] Folkman, J. (1971). Tumor angiogenesis: Therapeutic implications. New England Journal of Medicine, 285, 1182–1186. 4938153 10.1056/NEJM197111182852108

[brb3643-bib-0020] Glover, G. H. , & Law, C. S. (2001). Spiral‐in/out BOLD fMRI for increased SNR and reduced susceptibility artifacts. Magnetic Resonance in Medicine, 46, 515–522. 11550244 10.1002/mrm.1222

[brb3643-bib-0021] Harrison, N. A. , Doeller, C. F. , Voon, V. , Burgess, N. , & Critchley, H. D. (2014). Peripheral inflammation acutely impairs human spatial memory via actions on medial temporal lobe glucose metabolism. Biological Psychiatry, 76, 585–593. 24534013 10.1016/j.biopsych.2014.01.005 PMC4166523

[brb3643-bib-0022] He, B. J. (2011). Scale‐free properties of the functional magnetic resonance imaging signal during rest and task. The Journal of Neuroscience, 31, 13786–13795. 21957241 10.1523/JNEUROSCI.2111-11.2011 PMC3197021

[brb3643-bib-0023] He, B. J. , Zempel, J. M. , Snyder, A. Z. , & Raichle, M. E. (2010). The temporal structures and functional significance of scale‐free brain activity. Neuron, 66, 353–369. 20471349 10.1016/j.neuron.2010.04.020 PMC2878725

[brb3643-bib-0024] Hermundstad, A. M. , Brown, K. S. , Bassett, D. S. , & Carlson, J. M. (2011a). Architectural constraints on learning and memory function. BMC Neuroscience, 12, P31.

[brb3643-bib-0025] Hermundstad, A. M. , Brown, K. S. , Bassett, D. S. , & Carlson, J. M. (2011b). Learning, memory, and the role of neural network architecture. PLoS Computational Biology, 7, e1002063. 21738455 10.1371/journal.pcbi.1002063 PMC3127797

[brb3643-bib-0026] Honey, C. J. , Sporns, O. , Cammoun, L. , Gigandet, X. , Thiran, J. P. , Meuli, R. , & Hagmann, P. (2009). Predicting human resting‐state functional connectivity from structural connectivity. Proceedings of the National Academy of Sciences, 106, 2035–2040. 10.1073/pnas.0811168106 PMC2634800 19188601

[brb3643-bib-0027] Hosseini, S. M. , & Kesler, S. R. (2013). Comparing connectivity pattern and small‐world organization between structural correlation and resting‐state networks in healthy adults. NeuroImage, 78, 402–414. 23603348 10.1016/j.neuroimage.2013.04.032 PMC3673883

[brb3643-bib-0028] Hosseini, S. M. , Koovakkattu, D. , & Kesler, S. R. (2012). Altered small‐world properties of gray matter networks in breast cancer. BMC Neurology, 12, 28. 22632066 10.1186/1471-2377-12-28 PMC3404945

[brb3643-bib-0029] Hurst, H. (1951). Long‐term storage capacity of reservoirs. Trans Am Soc Civil Eng, 116, 770–808.

[brb3643-bib-0030] Janelsins, M. C. , Kesler, S. R. , Ahles, T. A. , & Morrow, G. R. (2014). Prevalence, mechanisms, and management of cancer‐related cognitive impairment. International Review of Psychiatry, 26, 102–113. 24716504 10.3109/09540261.2013.864260 PMC4084673

[brb3643-bib-0031] Jung, M. S. , Zhang, M. , Askren, M. K. , Berman, M. G. , Peltier, S. , Hayes, D. F. , … Cimprich, B. (2016). Cognitive dysfunction and symptom burden in women treated for breast cancer: a prospective behavioral and fMRI analysis. Brain Imaging and Behavior. doi:10.1007/s11682‐016‐9507‐8 10.1007/s11682-016-9507-8 26809289

[brb3643-bib-0032] Kesler, S. R. , Bennett, F. C. , Mahaffey, M. L. , & Spiegel, D. (2009). Regional brain activation during verbal declarative memory in metastatic breast cancer. Clinical Cancer Research, 15, 6665–6673. 19843664 10.1158/1078-0432.CCR-09-1227 PMC2859687

[brb3643-bib-0033] Kesler, S. R. , & Blayney, D. W. (2015). Neurotoxic effects of anthracycline‐ vs nonanthracycline‐based chemotherapy on cognition in breast cancer survivors. JAMA Oncology, 2, 1–8. 10.1001/jamaoncol.2015.4333 PMC4838415 26633037

[brb3643-bib-0034] Kesler, S. R. , Gugel, M. , Huston‐Warren, E. , & Watson, C. (2016). Atypical structural connectome organization and cognitive impairment in young survivors of acute lymphoblastic leukemia. Brain Connect, 6, 273–282. 26850738 10.1089/brain.2015.0409 PMC4876554

[brb3643-bib-0035] Kesler, S. R. , Gugel, M. , Pritchard‐Berman, M. , Lee, C. , Kutner, E. , Hosseini, S. M. , … Lacayo, N. (2014). Altered resting state functional connectivity in young survivors of acute lymphoblastic leukemia. Pediatric Blood & Cancer, 61, 1295–1299. 24619953 10.1002/pbc.25022 PMC4028071

[brb3643-bib-0036] Kesler, S. R. , Kent, J. S. , & O'Hara, R. (2011). Prefrontal cortex and executive function impairments in primary breast cancer. JAMA Neurology, 68, 1447–1453. 10.1001/archneurol.2011.245 PMC3239218 22084128

[brb3643-bib-0037] Kesler, S. R. , Watson, C. L. , & Blayney, D. W. (2015). Brain network alterations and vulnerability to simulated neurodegeneration in breast cancer. Neurobiology of Aging, 36, 2429–2442. 26004016 10.1016/j.neurobiolaging.2015.04.015 PMC4464941

[brb3643-bib-0038] Kesler, S. R. , Wefel, J. S. , Hosseini, S. M. , Cheung, M. , Watson, C. L. , & Hoeft, F. (2013). Default mode network connectivity distinguishes chemotherapy‐treated breast cancer survivors from controls. Proc Natl Acad Sci U S A, 110, 11600–11605. 23798392 10.1073/pnas.1214551110 PMC3710809

[brb3643-bib-0039] Koppelmans, V. , Vernooij, M. W. , Boogerd, W. , Seynaeve, C. , Ikram, M. A. , Breteler, M. M. , & Schagen, S. B. (2015). Prevalence of cerebral small‐vessel disease in long‐term breast cancer survivors exposed to both adjuvant radiotherapy and chemotherapy. Journal of Clinical Oncology, 33, 588–593. 25559803 10.1200/JCO.2014.56.8345

[brb3643-bib-0040] Kurth, F. , Gaser, C. , & Luders, E. (2015). A 12‐step user guide for analyzing voxel‐wise gray matter asymmetries in statistical parametric mapping (SPM). Nature Protocols, 10, 293–304. 25591011 10.1038/nprot.2015.014

[brb3643-bib-0041] Lepage, C. , Smith, A. M. , Moreau, J. , Barlow‐Krelina, E. , Wallis, N. , Collins, B. , … Scherling, C. (2014). A prospective study of grey matter and cognitive function alterations in chemotherapy‐treated breast cancer patients. Springerplus, 3, 444. 25184110 10.1186/2193-1801-3-444 PMC4149682

[brb3643-bib-0042] Mahalanobis, P. C. (1936). On the generalised distance in statistics. Proceedings National Institute of Science, India, 2, 49–55.

[brb3643-bib-0043] Manchon, J. F. , Dabaghian, Y. , Uzor, N. E. , Kesler, S. R. , Wefel, J. S. , & Tsvetkov, A. S. (2016). Levetiracetam mitigates doxorubicin‐induced DNA and synaptic damage in neurons. Scientific Reports, 6, 25705. 27168474 10.1038/srep25705 PMC4863375

[brb3643-bib-0044] McDonald, B. C. , Conroy, S. K. , Ahles, T. A. , West, J. D. , & Saykin, A. J. (2012). Alterations in brain activation during working memory processing associated with breast cancer and treatment: A prospective functional magnetic resonance imaging study. Journal of Clinical Oncology, 30, 2500–2508. 22665542 10.1200/JCO.2011.38.5674 PMC3397784

[brb3643-bib-0045] Mechelli, A. , Friston, K. J. , Frackowiak, R. S. , & Price, C. J. (2005). Structural covariance in the human cortex. Journal of Neuroscience, 25, 8303–8310. 16148238 10.1523/JNEUROSCI.0357-05.2005 PMC6725541

[brb3643-bib-0046] Menning, S. , de Ruiter, M. B. , Veltman, D. J. , Koppelmans, V. , Kirschbaum, C. , Boogerd, W. , … Schagen, S. B. (2015). Multimodal MRI and cognitive function in patients with breast cancer prior to adjuvant treatment—The role of fatigue. NeuroImage Clinical, 7, 547–554. 25844311 10.1016/j.nicl.2015.02.005 PMC4375788

[brb3643-bib-0047] Moses, J. (2004). Comprehensive Trail Making Test (CTMT)By Cecil R. Reynolds. Austin, Texas: PRO‐ED Inc, 2002. Archives of Clinical Neuropsychology, 19, 703–708. 15330000 10.1016/j.acn.2004.02.004

[brb3643-bib-0048] Ng, T. , Cheung, Y. T. , Ham Guo, M. S. , Kee, Y. C. , Ho, H. K. , Fan, G. , … Chan, A. (2013). Plasma vascular endothelial growth factor level and cognitive changes in breast cancer patients. Journal of Clinical Oncology (Meeting Abstracts), 31, e20566.

[brb3643-bib-0049] Nudelman, K. N. , Wang, Y. , McDonald, B. C. , Conroy, S. K. , Smith, D. J. , West, J. D. , … Saykin, A. J. (2014). Altered cerebral blood flow one month after systemic chemotherapy for breast cancer: A prospective study using pulsed arterial spin labeling MRI perfusion. PLoS One, 9, e96713. 24816641 10.1371/journal.pone.0096713 PMC4016018

[brb3643-bib-0050] Palop, J. J. , & Mucke, L. (2010). Synaptic depression and aberrant excitatory network activity in Alzheimer's disease: Two faces of the same coin? Neuromolecular Medicine, 12, 48–55. 19838821 10.1007/s12017-009-8097-7 PMC3319077

[brb3643-bib-0051] Patel, A. X. , Kundu, P. , Rubinov, M. , Jones, P. S. , Vertes, P. E. , Ersche, K. D. , Suckling, J. , & Bullmore, E. T. (2014). A wavelet method for modeling and despiking motion artifacts from resting‐state fMRI time series. NeuroImage, 95, 287–304. 24657353 10.1016/j.neuroimage.2014.03.012 PMC4068300

[brb3643-bib-0052] Patel, S. K. , Wong, A. L. , Wong, F. L. , Breen, E. C. , Hurria, A. , Smith, M. , … Bhatia, S. (2015). Inflammatory Biomarkers, Comorbidity, and Neurocognition in Women With Newly Diagnosed Breast Cancer. Journal of the National Cancer Institute, 107, 1–7. 10.1093/jnci/djv131 PMC4609551 26101331

[brb3643-bib-0053] Pundavela, J. , Roselli, S. , Faulkner, S. , Attia, J. , Scott, R. J. , Thorne, R. F. , … Hondermarck, H. (2015). Nerve fibers infiltrate the tumor microenvironment and are associated with nerve growth factor production and lymph node invasion in breast cancer. Molecular Oncology, 9, 1626–1635. 26009480 10.1016/j.molonc.2015.05.001 PMC5528785

[brb3643-bib-0054] Roth, R. M. , Isquith, P. K. , & Gioia, G. (2005). Behavioral Rating Inventory of Executive Function ‐ Adult Version. Lutz, FL: Psychological Assessment Resources.

[brb3643-bib-0055] Rubinov, M. , & Sporns, O. (2010). Complex network measures of brain connectivity: Uses and interpretations. NeuroImage, 52, 1059–1069. 19819337 10.1016/j.neuroimage.2009.10.003

[brb3643-bib-0056] Rudie, J. D. , Brown, J. A. , Beck‐Pancer, D. , Hernandez, L. M. , Dennis, E. L. , Thompson, P. M. , … Dapretto, M. (2012). Altered functional and structural brain network organization in autism. NeuroImage Clinical, 2, 79–94. 24179761 10.1016/j.nicl.2012.11.006 PMC3777708

[brb3643-bib-0057] Ruff, R. M. , Light, R. H. , Parker, S. B. , & Levin, H. S. (1996). Benton Controlled Oral Word Association Test: Reliability and updated norms. Arch Clin Neuropsychol, 11, 329–338. 14588937

[brb3643-bib-0058] de Ruiter, M. B. , Reneman, L. , Boogerd, W. , Veltman, D. J. , van Dam, F. S. , Nederveen, A. J. , … Schagen, S. B. (2011). Cerebral hyporesponsiveness and cognitive impairment 10 years after chemotherapy for breast cancer. Human Brain Mapping, 32, 1206–1219. 20669165 10.1002/hbm.21102 PMC6869999

[brb3643-bib-0059] Sato, C. , Sekiguchi, A. , Kawai, M. , Kotozaki, Y. , Nouchi, R. , Tada, H. , … Ohuchi, N. (2015). Postoperative structural brain changes and cognitive dysfunction in patients with breast cancer. PLoS One, 10, e0140655. 26536672 10.1371/journal.pone.0140655 PMC4633203

[brb3643-bib-0060] Scherling, C. , Collins, B. , Mackenzie, J. , Bielajew, C. , & Smith, A. (2011). Pre‐chemotherapy differences in visuospatial working memory in breast cancer patients compared to controls: An FMRI study. Frontiers in Human Neuroscience, 5, 122. 22053153 10.3389/fnhum.2011.00122 PMC3205481

[brb3643-bib-0061] Scherling, C. , Collins, B. , Mackenzie, J. , Bielajew, C. , & Smith, A. (2012). Prechemotherapy differences in response inhibition in breast cancer patients compared to controls: A functional magnetic resonance imaging study. Journal of Clinical and Experimental Neuropsychology, 34, 543–560. 22380580 10.1080/13803395.2012.666227

[brb3643-bib-0062] Scherling, C. , Collins, B. , MacKenzie, J. , Lepage, C. , Bielajew, C. , & Smith, A. (2012). Structural brain differences in breast cancer patients compared to matched controls prior to chemotherapy. International Journal of Biology, 4.

[brb3643-bib-0063] Schmidt, M. (2012). Rey auditory verbal learning test (RAVLT): A handbook. Lutz, FL: Psychological Assessment Resources.

[brb3643-bib-0064] Simo, M. , Root, J. C. , Vaquero, L. , Ripolles, P. , Jove, J. , Ahles, T. , … Rodriguez‐Fornells, A. (2015). Cognitive and brain structural changes in a lung cancer population. Journal of Thoracic Oncology, 10, 38–45. 25325778 10.1097/JTO.0000000000000345 PMC5657249

[brb3643-bib-0065] Sporns, O. , & Honey, C. J. (2006). Small worlds inside big brains. Proceedings of National Academy of Sciences United States of America, 103, 19219–19220. 10.1073/pnas.0609523103 PMC1748207 17159140

[brb3643-bib-0066] Stam, C. J. , van Straaten, E. C. , Van Dellen, E. , Tewarie, P. , Gong, G. , Hillebrand, A. , … Van Mieghem, P. (2016). The relation between structural and functional connectivity patterns in complex brain networks. International Journal of Psychophysiology, 103, 149–160. 25678023 10.1016/j.ijpsycho.2015.02.011

[brb3643-bib-0067] Stouten‐Kemperman, M. M. , de Ruiter, M. B. , Boogerd, W. , Veltman, D. J. , Reneman, L. , & Schagen, S. B. (2014). Very late treatment‐related alterations in brain function of breast cancer survivors. Journal of the International Neuropsychological Society, 21, 1–12. 25529014 10.1017/S1355617714001015

[brb3643-bib-0068] Stouten‐Kemperman, M. M. , de Ruiter, M. B. , Caan, M. W. , Boogerd, W. , Kerst, M. J. , Reneman, L. , & Schagen, S. B. (2015). Lower cognitive performance and white matter changes in testicular cancer survivors 10 years after chemotherapy. Human Brain Mapping, 36, 4638–4647. 26304182 10.1002/hbm.22942 PMC6869574

[brb3643-bib-0069] Tijms, B. M. , Series, P. , Willshaw, D. J. , & Lawrie, S. M. (2012). Similarity‐based extraction of individual networks from gray matter MRI scans. Cerebral Cortex, 22, 1530–1541. 21878484 10.1093/cercor/bhr221

[brb3643-bib-0070] Tzourio‐Mazoyer, N. , Landeau, B. , Papathanassiou, D. , Crivello, F. , Etard, O. , Delcroix, N. , … Joliot, M. (2002). Automated anatomical labeling of activations in SPM using a macroscopic anatomical parcellation of the MNI MRI single‐subject brain. NeuroImage, 15, 273–289. 11771995 10.1006/nimg.2001.0978

[brb3643-bib-0071] Wefel, J. S. , Kesler, S. R. , Noll, K. R. , & Schagen, S. B. (2015). Clinical characteristics, pathophysiology, and management of noncentral nervous system cancer‐related cognitive impairment in adults. CA: A Cancer Journal for Clinicians, 65, 123–138. 25483452 10.3322/caac.21258 PMC4355212

[brb3643-bib-0072] Wirsich, J. , Perry, A. , Ridley, B. , Proix, T. , Golos, M. , Benar, C. , … Guye, M. (2016). Whole‐brain analytic measures of network communication reveal increased structure‐function correlation in right temporal lobe epilepsy. Neuroimage Clin, 11, 707–718. 27330970 10.1016/j.nicl.2016.05.010 PMC4909094

[brb3643-bib-0073] Zhao, Q. , Yang, Y. , Liang, X. , Du, G. , Liu, L. , Lu, L. , … Zhang, G. (2014). The clinicopathological significance of neurogenesis in breast cancer. BMC Cancer, 14, 1–6. 24996968 10.1186/1471-2407-14-484 PMC4107959

